# Correlation Between Serum Concentrations of Menaquinone-4 and Developmental Quotients in Children With Autism Spectrum Disorder

**DOI:** 10.3389/fnut.2021.748513

**Published:** 2021-09-30

**Authors:** Hanyu Dong, Bing Wang, Junyan Feng, Xiaojing Yue, Feiyong Jia

**Affiliations:** Department of Developmental and Behavioral Pediatrics, The First Hospital of Jilin University, Changchun, China

**Keywords:** ASD, developmental quotients, vitamin K, menaquinone-4, cognition

## Abstract

**Objective:** The vitamin K family has a wide range of effects in the body, including the central nervous system. Menaquinone-4 (MK-4), a form of vitamin K2, is converted from phylloquinone (PK), which is the main source of dietary vitamin K and is the main form of vitamin K in the brain. We conducted this study to investigate the serum concentration of MK-4 and the correlations between MK-4 and developmental quotients in children with autism spectrum disorder (ASD).

**Methods:** We selected 731 children with ASD who were diagnosed for the first time. During the same period, 332 neurotypical children who underwent regular physical examinations in our outpatient department were selected as the TD group. We investigated the general situation of children, including gender and age. Children in ASD group were assessed for autistic symptoms and development quotients, including Autism Behavior Checklist (ABC), Childhood Autism Rating Scale (CARS), ADOS-2, and Griffiths Development Scales-Chinese Language Edition (GDS-C). Both groups of children were tested for serum menaquinone-4. We compared serum menaquinone-4 levels of ASD group and TD group. We then conducted a correlation analysis between the level of menaquinone-4 and the developmental quotient of children with ASD.

**Results:** The results of this study indicate that the serum concentration of MK-4 in children with ASD is lower than that in children with typical development (*t* = −2.702, *P* = 0.007). The serum concentration of MK-4 is related to the developmental quotients of several subscales in ASD children, and this correlation is more obvious in males.

**Conclusion:** we conclude that MK-4 is present in lower concentrations in children with ASD, which may affect cognition and developmental quotients. The role of MK-4 in ASD needs to be further explored.

## Introduction

Vitamin K is a fat-soluble vitamin. The vitamin K family contains a 2-methyl-1,4- naphthoquinone ring, but their structure at the 3-position is not the same (1). According to the structure of the 3-position, vitamin K exists in two forms. Vitamin K1 is phylloquinone (2-methyl-3-phytyl-1,4-naphthoquinone) (PK), which is synthesized in plants and is the main source of dietary vitamin K (1). Vitamin K2 is the menaquinones. According to the amount of prenyl contained in it, vitamin K2 is further classified as menadione-n (MK-6~13). These molecules are synthesized by bacteria and are the main form of vitamin K in animal tissues. Notably, menaquinone-4 (MK-4) in vitamin K2 is a non-bacterial menaquinone. MK-4 is converted from PK in the body under the catalysis of UbiA prenyltransferase containing 1 (UBIAD1) (2). It is commonly found in extrahepatic tissues of animals and is the main form of vitamin K in the brain (3, 4). Animal experiments have shown that in rats, 98% of vitamin K in the brain is MK-4 (5).

The vitamin K family has a wide range of effects in the body. In addition to its effect on blood coagulation, bones and the cardiovascular system, an increasing number of studies have begun to pay attention to the role of vitamin K in tumors (6) and the nervous system (7). However, compared to the effect of vitamin K on other systems, its effect on the nervous system has been overlooked to some extent, especially the effect of MK-4.

The effects of MK-4 on the brain are mainly reflected in the following aspects. First, early studies have shown that MK-4 can promote sphingolipid synthesis (8, 9). The role of sphingolipids is not only that of a structural component of the cell membrane, but sphingolipids also have a close connection with cell proliferation, differentiation, senescence, cell-cell interactions, and transformation, and even degenerative diseases of the nervous system (10–13). Second, two vitamin K-dependent proteins play an important role in the nervous system, namely, Gas6 and protein S. The two proteins share 44% amino acid homology in structure (14). Gas6, which is widely expressed in the central nervous system, has a wide range of effects on nerve development and participates in cell survival, growth, myelination, and other functions (15). It can also inhibit tumor necrosis factor alpha (TNF-α) and its toxic effect on oligodendrocytes (16). In the past, it was thought that protein S was used as a cofactor of protein C to play a role in the coagulation process (17). However, protein S also has a neuroprotective effect, which mainly protects the brain through antithrombotic effects and signal-mediated neuroprotection (18). Third, there are still studies that have found that MK-4 has a protective effect against oxidative stress and inflammation (19), and this effect may be accomplished by preventing glutathione depletion and inhibiting proinflammatory markers (20, 21).

Autism spectrum disorder (ASD) was originally defined by Leo Kanner (22), and is characterized by persistent deficits in social communication and interaction and stereotyped or repetitive patterns of behavior, interests or activities. ASD is classified as a neurodevelopmental disorder in the Diagnostic and Statistical Manual of Mental Disorders 5th Edition (DSM-5). During the last few decades, the reported prevalence of autism in children has increased dramatically. The latest data (23) released by the Centers for Disease Control and Prevention in the United States in 2020 shows that across all 11 sites in American, ASD prevalence was 18.5 per 1,000 (one in 54) children aged 8 years. It has led to the development of many social issues and placed a heavy burden on families. Unfortunately, we have not yet fully unveiled the mystery of autism. At present, the exact cause and cure of ASD are not yet known.

At present, it is believed that ASD is the result of the combined effects of genetic and environmental factors, and an important aspect of the environmental factors of ASD is nutritional factors. In view of the aforementioned important role of vitamin K in the nervous system, we believe that it is of great significance to study the nutritional status of vitamin K in children with ASD. At present, studies have shown that children with autism have some problems with vitamin K intake (24). Adams' research shows that vitamin K levels in children with ASD are related to the severity of symptoms (25).

However, very few studies have focused on the relationship between vitamin K levels and ASD in children. We conducted this study based on the theoretical basis above, aiming to focus on the serum concentration of MK-4 and the correlation between MK-4 and the developmental quotient (DQ) in children with ASD. This may lay the foundation for future research on the role of vitamin K in ASD.

## Methods

### Participants

We selected 731 children with ASD who were diagnosed for the first time in the Department of Developmental and Behavioral Pediatrics of the First Hospital of Jilin University between July 2020 and January 2021 as the ASD group. During the same period, 332 neurotypical children who underwent regular physical examinations in our outpatient department were selected as the TD group. The age of the ASD group was 44.88 ± 19.13 months, and that of the TD group was 47.53 ± 36.537 months. The sex and age comparisons between the two groups were not statistically significant (*P* = 0.733 and 0.124, respectively; Table 1). The inclusion criteria for the children with ASD were that the Autism Diagnostic Observation Schedule-Second Edition (ADOS-2) was used to diagnose the children for the first time without systemic intervention. The inclusion criteria for the children with typical development were as follows: children undergoing routine physical examinations in outpatient clinics; children with height and weight development within the normal range; children with a DQ above 85 in all domains of the Griffiths Development Scales-Chinese Language Edition (GDS-C); and children whose outpatient doctors' mental examination clearly indicated that they do not meet the ASD or other neurodevelopmental disorders' diagnostic criteria in the DSM-5. The Exclusion criteria include malabsorption syndromes, abnormal liver function, bleeding tendency, bone, and cardiovascular system diseases. Long-term use of broad-spectrum antibiotics, multivitamins (especially fat-soluble vitamins, like vitamin A, D, and E), warfarin, and other drugs that may affect the metabolism of vitamin K in the body in the past 6 months should also be excluded. Children with severe physical disability, or uncontrolled epilepsy were also excluded. The study protocol was approved by the ethics committee of our hospital, and informed consent was provided by the parents or caregivers of the children who participated.

**Table 1:** Comparison of menaquinone-4 levels between the ASD group and the TD group.

	**ASD group**	**TD group**	**t/X2**	***P***
**Age**	44.88 ± 19.13	47.53 ± 36.537	−1.540	0.124
**Sex**				
Male	603	271	0.116	0.733
Female	128	61		
**Menaquinone-4**	0.123 ± 0.167	0.155 ± 0.208	−2.702	0.007^*^

* *P < 0.05*.

### Procedure

We investigated the general characteristics of children, including sex and age. Children in the ASD group were assessed for autistic symptoms and the DQ, including the Autism Behavior Checklist (ABC), Childhood Autism Rating Scale (CARS), ADOS-2, and GDS-C. Both groups of children were tested for serum MK-4.

We compared the age, sex-related rate, and serum MK-4 levels of the ASD group and TD group. The concentration of MK-4 was divided into three levels: deficient, sufficient and excess levels, and the distribution of MK-4 levels between the two groups was compared. The ASD group and the TD group were divided into male and female subgroups according to sex. We compared the MK-4 levels of different sexes in the ASD group and the TD group and then compared vitamin K levels of the same sex between the two groups.

We then conducted a correlation analysis between the level of MK-4 and the DQ of children with ASD. The ASD group was further divided into two subgroups according to sex, and the aforementioned correlation analysis was performed.

The ADOS-2 was utilized in this study as a diagnostic tool for ASD. The ADOS-2 is a semistructured, standardized assessment tool for individuals with suspected ASD that measures autism symptoms in social relatedness, communication, play, and repetitive behaviors and is deemed to be part of the gold standard for ASD diagnostic evaluation (26). ADOS-2 modules 1 and 2 have calibrated severity scores such that 3–4 is low-level evidence, 5–7 is moderate-level evidence, and 8–10 is high-level evidence. The ADOS-2 Module Toddler, which is used for children aged under 31 months, has no calibrated severity scores.

The GDS-C is a popular tool in the Chinese social context and has good reliability and validity (27). It uses five independent subscales to assess the development level of children aged 0–2 years: physical mobility (A scale), personal social skills (B scale), hearing-speech (C scale), eye-hand coordination (D scale), and performance (E scale). Children aged 3–8 have increased practical reasoning (F scale). The test scores were converted to developmental age (DA) according to the Chinese norm for the GDS-C; chronological age (CA) was calculated as the date of assessment minus the date of birth; and DQ = DA × 100/CA (28). The DQ of each scale is called the AQ, BQ, CQ, DQ, EQ, and FQ.

### Statistical Methods

SPSS 23.0 statistical software was used for statistical analysis. Normally distributed measurement data are expressed as the mean ± standard deviation (SD). Two independent-samples *t*-tests were used for comparisons between the groups. The enumeration data are presented as *N* (%), and the differences between the two groups were measured by chi-square tests. Spearman rank correlation was used for the two non-normally distributed variables of the correlation study.

## Results

1. Comparison of MK-4 levels between the ASD group and the TD group

The level of MK-4 in the ASD group was 0.123 ± 0.167 ng/ml, and the level of MK-4 in the TD group was 0.155 ± 0.208 ng/ml. The comparison between the two groups was statistically significant (*t* = −2.702, *P* = 0.007; Table 1).

The MK-4 levels of the two groups were divided into three levels, namely, deficient, sufficient and excess, and the comparison of the rates between the two groups was statistically significant (*X*^2^ = 15.209, *P* = 0.000; Table 2).

**Table 2:** Comparison of the distribution of menaquinone-4 levels between the two groups.

	**ASD group**	**TD group**	***X* ^**2**^**	***P***
<0.1 ng/ml	443	159	15.209	0.000^*^
0.1–0.86 ng/ml	280	167		
>0.86 ng/ml	8	6		

* *P < 0.05*.

The MK-4 levels between the sexes in the ASD group and the TD group were not statistically significant (ASD group, *t* = 1.247, *P* = 0.213; TD group, *t* = 1.060, *P* = 0.290). The MK-4 level of males between the two groups was statistically significant (*t* = −2.487, *P* = 0.013). The MK-4 level of females between the two groups was not significantly different (*t* = 1.060, *P* = 0.290; Table 3).

**Table 3:** Comparison of menaquinone-4 levels between males and females in the two groups.

	**Male**	**Female**	***t***	***P***
ASD group	0.126 ± 0.171	0.106 ± 0.148	1.247	0.213
TD group	0.161 ± 0.226	0.129 ± 0.083	1.060	0.290
*t*	−2.487	−1.161		
*P*	0.013^*^	0.247		

* *P < 0.05*.

2. Correlation analysis of MK-4 levels and DQs in children with ASD

The level of MK-4 in the ASD group had a positive correlation with the DQs of physical mobility (*r* = 0.110, *P* = 0.007), personal social skills (*r* = 0.117, *P* = 0.004), hearing-speech (*r* = 0.100, *P* = 0.014), and eye-hand coordination (*r* = 0.083, *P* = 0.040). There was no obvious correlation with the DQs of performance and practical reasoning (*P* = 0.184 and 0.907, respectively; Table 4).

**Table 4:** Correlation of menaquinone-4 levels and DQs in children with ASD.

	**AQ**	**BQ**	**CQ**	**DQ**	**EQ**	**FQ**
*r*	0.110	0.117	0.100	0.083	0.054	-0.015
*P*	0.007^*^	0.004^*^	0.014^*^	0.040^*^	0.184	0.907

* *P <0.05*.

In the male subgroup of the ASD group, the level of MK-4 had a positive correlation with the DQs of physical mobility (*r* = 0.100, *P* = 0.026), personal social skills (*r* = 0.131, *P* = 0.003), and hearing-speech (*r* = 0.109, *P* = 0.015) and had no obvious correlation with the DQs of eye-hand coordination, performance, and practical reasoning (*P* = 0.051, 0.153, and 0.895, respectively; Table 5). In the female subgroup of the ASD group, there was no significant correlation between the level of MK-4 and the DQs of each subscale (*P* = 0.096, 0.759, 0.481, 0.473, 0.995, and 0.145, respectively; Table 5).

**Table 5:** Correlation of menaquinone-4 levels and DQs in male and female subgroups.

		**AQ**	**BQ**	**CQ**	**DQ**	**EQ**	**FQ**
Male	*r*	0.100	0.131	0.109	0.087	0.064	0.018
	*P*	0.026^*^	0.003^*^	0.015^*^	0.051	0.153	0.895
Female	*r*	0.161	0.030	0.068	0.070	0.001	−0.565
	*P*	0.096	0.759	0.481	0.473	0.995	0.145

* *P < 0.05*.

## Discussion

The results of this study indicate that the serum concentration of MK-4 in children with ASD is lower than that in children with typical development. The serum concentration of MK-4 is related to the DQ of several subscales in ASD children, and this correlation is more obvious in males.

1. Serum concentrations of MK-4 in children with ASD

Our study found that the serum concentration of MK-4 in children with ASD was lower. There are no relevant studies that have similar conclusions, but studies have shown that the vitamin K intake in children with ASD is insufficient, which may result in insufficient blood MK-4 levels. In Lindsay's study (24), the baseline level of nutritional intake of 20 children with ASD aged 5–13 was investigated, and 9 of the 20 children with ASD had insufficient dietary nutrient reference intake (DRI) levels of vitamin K. Other nutrients that are lower than the DRI but represented in a high proportion of the children included vitamin D (10/20) and Ca (10/20). Vitamin D, vitamin K, and calcium have a certain synergistic effect on bone metabolism, which also prompts us to pay attention to the interactions among them in neurodevelopment.

Another point worth noting is that vitamin K is a fat-soluble vitamin that can be distributed in adipose tissue, and studies (29) have shown that men and women with higher percentage body fat had poorer vitamin K status (although it is adult research data). While ASD Children have a higher proportion of overweight and obesity (30), and the intake of vitamin K may be insufficient (24), which may also be the cause of lower vitamin K levels in children with ASD. The relationship between body mass index, vitamin K intake, and vitamin K level of children with ASD is worthy of further discussion and verification.

The serum concentration of vitamin K may be related to sex. Our research shows that in males, the concentration of MK-4 in the ASD group was lower than that in the TD group. In females, the concentration of MK-4 in the ASD group seemed to be slightly lower than that in the TD group, but there was no significant difference between the two groups. There are still no relevant studies that have the same conclusion. However, there have been related studies on the relationship between vitamin K intake and sex.

The latest research by Alkhalidy et al. (31) shows that autistic boys were at higher risk of inadequate vitamin K status than TD children. The proportion of boys with ASD with insufficient vitamin K intake was 86.1%, which was significantly higher than that of boys in the TD group (61.5%). Regarding the differences in vitamin K between the sexes, animal experiments (32) have found that with the same diet, male rats are more likely to be deficient in vitamin K than female rats and are more susceptible to the adverse effects of low vitamin K. There are also related studies on the MK-4 subgroup of vitamin K (5). The concentration of MK-4 in the brains of female rats was higher than that in the brains of male rats, and it decreased with age and increased with increasing phytoquinone intake. As a vitamin K antagonist, warfarin can inhibit the coagulation process involved in vitamin K. At present, studies (33–35) have shown that the anticoagulant process of warfarin is different between men and women, and men have a higher risk of bleeding. This phenomenon may also be related to the metabolism of vitamin K in different genders, suggesting that men are more susceptible to the adverse effects of vitamin K function inhibition. Therefore, combined with the results of our study, we have reason to infer that it may have similar effects on the nervous system of ASD children of different sexes. This may also be one of the underlying factors that cause the different prevalence of ASD between boys and girls.

2. The relationship between MK-4 and the DQs in children with ASD

Our study suggested that the concentration of MK-4 in ASD children has a positive correlation with the subscales of physical mobility (A scale), personal social skills (B scale), hearing-speech (C scale), and eye-hand coordination (D scale). The higher the concentration of MK-4, the better is the DQ. This correlation can still be observed in boys but does not exist in girls.

At present, research on the development of ASD in children is temporarily lacking, but it is not uncommon for researchers to examine the correlations between vitamin K and cognition as well as behavior, regardless of whether the brain of children or adults is being studied. Pauli (36, 37) showed that exposure to the vitamin K inhibitor warfarin can cause brain damage during the embryonic period and can cause intellectual disability. In adult patients with Alzheimer's disease (38), it was also found that early-stage Alzheimer's patients consumed less vitamin K than the cognitively normal control group. In elderly individuals with memory loss, increasing the intake of dietary vitamin K has a positive effect on improving cognitive symptoms such as memory loss (39). Cocchetto's animal experiments (40) also show that a vitamin-K-deficient diet or the application of warfarin can cause decreased activity and decreased exploration ability in rats. However, there are also studies (41) suggesting that cerebral MK-4 has nothing to do with cognition. Further investigation of vitamin K uptake and metabolism in the brain is warranted.

3. The possible role of MK-4 in the neurodevelopment of children with ASD

The relevant evidence for the role of MK-4 in the nervous system has been described above. Based on the results of this study and the role of MK-4 in the nervous system, we speculate that the role of MK-4 in ASD may be as follows.

First, the vitamin-K-dependent protein Gas6 can inhibit TNF-α and oligodendrocyte activity (16). Studies (42, 43) have shown that TNF-α in ASD patients is significantly elevated in various tissues. Moreover, the expression of TNF-α mRNA in children with ASD is also related to sex, which may be related to the sex ratio of ASD (44). In addition, oligodendrocytes may disrupt neurotransmission or modify axonal conduction to cause neurochemical imbalance in the brains of ASD patients (45). In summary, we believe that vitamin K can act as a protective factor in the pathogenesis of ASD by inhibiting the activity of TNF-α and oligodendrocytes.

Second, it can protect the body from oxidative stress and promote the formation of nerve myelin. Yui's research (46) shows that oxidative stress can participate in the pathophysiological process of ASD by affecting the process of myelination. Oxidative free radicals can disrupt the myelination process (47). In particular, oligodendrocytes are very susceptible to oxidative free radicals (48) because they have lower glutathione levels and a higher sphingolipid content (48). On the one hand, vitamin K can prevent glutathione depletion and protect immature cortical neurons, thereby blocking the damage from oxidative stress on developing oligodendrocytes (19). On the other hand, vitamin K can promote sulfide in the brain. This production directly promotes the formation of the myelin sheath (49).

## Limitations

- Vitamin K and vitamin D have a certain synergistic effect on bone metabolism and the cardiovascular system. Research on the role of vitamin D in ASD has received widespread attention. This study focuses only on the concentration of vitamin K in children with ASD and its correlation with development. However, attention was not paid to the possible synergy of vitamin D and vitamin K in ASD patients. This is the direction of future research.
- MK-4 is a type of vitamin K2 derived from PK, and its concentration is closely related to diet. This study has not yet tracked and compared the intake of vitamin K in the two groups of children. Follow-up studies will focus on this part.
- This study did not focus on the relationship between the concentration of MK-4 and the core symptoms of autism, which deserves further research and will provide a basis for further exploring the role of MK-4 in children with ASD.

## Conclusion

MK-4 is present in lower concentrations in children with ASD, which may affect cognition and is related to the DQ, and this correlation may be related to sex. The role of MK-4 in ASD needs to be further explored.

## Data Availability Statement

The raw data supporting the conclusions of this article will be made available by the authors, without undue reservation.

## Ethics Statement

The studies involving human participants were reviewed and approved by the Ethics Committee of First Hospital of Jilin University. Written informed consent to participate in this study was provided by the participants' legal guardian/next of kin.

## Author Contributions

HD: analysis and interpretation of data, drafting the article, and investigation. BW: analysis data and drafting the article (tables). JF: supervising, editing, and clinical trial registration. XY: acquisition of data. FJ: concept and design, revising the article critically for important intellectual content, final approval of the version to be published, and funding acquisition. All authors contributed to the article and approved the submitted version.

## Funding

This work was supported by the National Natural Science Foundation of China (Grant No. 81973054), Key Scientific and Technological Projects of Guangdong Province (Grant No. 2018B030335001), Joint Fund Bethune Medical Special Project of Jilin Province (Grant No. 20200201507JC), and the Project of Jilin Provincial Department of Finance (Grant No. 2018SCZWSZX-60).

## Conflict of Interest

The authors declare that the research was conducted in the absence of any commercial or financial relationships that could be construed as a potential conflict of interest.

## Publisher's Note

All claims expressed in this article are solely those of the authors and do not necessarily represent those of their affiliated organizations, or those of the publisher, the editors and the reviewers. Any product that may be evaluated in this article, or claim that may be made by its manufacturer, is not guaranteed or endorsed by the publisher.
